# The traps of adaptation: Addiction as maladaptive referent-dependent evaluation

**DOI:** 10.3758/s13415-023-01086-4

**Published:** 2023-04-04

**Authors:** Francesco Rigoli, Giovanni Pezzulo

**Affiliations:** 1 Department of Psychology, City, University of London, Northampton Square, London, EC1V 0HB UK; 2 Institute of Cognitive Sciences and Technologies, National Research Council of Italy, Rome, Italy

**Keywords:** Addiction, Referent dependent, Evaluation, Reference point, Context

## Abstract

Referent-dependent evaluation theories propose that the ongoing context influences how the brain attributes value to stimuli. What are the implications of these theories for understanding addiction? The paper asks this question by casting this disorder as a form of maladaptive referent-dependent evaluation. Specifically, addiction is proposed to arise from the establishment of an excessive reference point following repeated drug consumption. Several key aspects of the disorder emerge from this perspective, including withdrawal, tolerance, enhanced craving, negative mood, and diminished stimulus discriminability. As highlighted in the paper, this formulation has important analogies with classical accounts of addiction, such as set point theories and associative learning theories. Moreover, this picture fits with the pattern of striatal dopaminergic activity observed in addiction, a key neural signature of the disorder. Overall, the referent-dependent evaluation approach emerges as a useful add-on to the theoretical toolkit adopted to interpret addiction. This also supports the idea that referent-dependent evaluation might offer a general framework to understand various disorders characterised by disrupted motivation.

## Introduction

Addiction is a neuropsychological disorder characterized by compulsive use of a drug despite severe adverse consequences (West & Brown, 2013). The impact of this condition upon society is dramatic, both in terms of health and economic consequences (Peacock et al., 2018). Considering the United States as an example, it is estimated that addiction accounts for up to 20% deaths each year and for one-third of inpatient hospital costs (Galanter et al., 2015). Understanding the neuropsychological processes underlying the development and maintenance of this disorder is therefore of paramount importance.

It is widely recognised that disrupted evaluation is at the root of addiction (West & Brown, 2013). Evaluation can be defined as the process whereby the brain attributes incentive value to stimuli, thereby establishing to what extent these stimuli are to be sought or avoided. The critical role of evaluation in addiction is evident from the fact that an excessive desire for a drug (i.e., craving) is at the core of this disorder (West & Brown, 2013). Moreover, aberrant evaluation appears not only to target the addictive substance, but also other stimuli, as evidenced by the fact that the ability to weight actions’ costs and benefits seems often to be impaired in addicted patients (Clark & Robbins, 2002; Koffarnus & Kaplan, 2018; Verdejo-Garcia et al., 2018). Based on this argument, examining the implications of contemporary neuropsychological theories of evaluation for addiction appears as a promising research endeavour. These theories highlight the referent-dependent nature of evaluation, namely the notion that value is not attributed in a vacuum, but within a specific context that shapes the process in a fundamental manner (Kőszegi & Rabin, 2006; Louie et al., 2015; Rigoli, 2019; Rigoli et al., 2016; Stewart et al., 2006). The idea of referent-dependent evaluation can be traced back to the introduction of Prospect Theory (Kahneman & Tversky, 1979), proposing that the status quo corresponds to a reference point in comparison to which outcomes are judged as either losses or gains. Building upon this seminal framework, contemporary theories interpret evaluation as being shaped by the distribution of stimuli one associates with the ongoing environment or context (Kőszegi & Rabin, 2006; Louie et al., 2015; Rigoli, 2019; Rigoli et al., 2016; Stewart et al., 2006; Woodford, 2012). What does this view imply for understanding addiction? Can any new insight be gained by framing addiction as a form of abnormal referent-dependent evaluation? The present paper addresses these questions. The next section overviews contemporary theories of referent-dependent evaluation. It will be followed by a section that proposes to interpret addiction as a form of maladaptive referent-dependent evaluation. Next, the paper will examine analogies between this interpretation and classical models of addiction, encompassing an analysis of both psychological and neural facets. We will conclude by considering broader implications of adopting this new perspective.

### Theories of referent-dependent evaluation

Contemporary literature presents three main research frameworks where the notion of referent-dependent evaluation is pivotal. Influential in psychology, decision-by-sampling theory postulates that, each time a new stimulus is encountered, representations of stimuli experienced in the past are sampled from memory (Stewart et al., 2006). According to this theory, a comparison between the ongoing stimulus and memory samples is the process through which value is attributed to the stimulus. Because which samples are retrieved from memory depends on the ongoing context (e.g., on the recent distribution of stimuli), decision-by-sampling implies that the context plays a critical role in shaping evaluation. Popular in cognitive neuroscience, divisive normalization theory is a second framework emphasising referent-dependent processes in evaluation (Louie et al., 2015). The key assumption of this perspective is that efficient coding drives the functioning of the brain. Efficient coding prescribes that, because of a limited range of spiking rate, neurons must tune their response sensitivity to the recent distribution of stimuli. Applying this principle to evaluation implies that the value attributed to a stimulus will critically depend on the ongoing context. Expectation-as-reference is a third framework aiming at explaining referent-dependent evaluation (Kőszegi & Rabin, 2006; Rigoli et al., 2016). Here, the assumption is that the brain explicitly represents statistics (such as the mean and standard deviation) describing the recent distribution of stimuli and relies on these statistics to evaluate a new stimulus. Thus, a key role for the context also ensues from this perspective.

Despite important differences, the theories described above share a similar logic and implicate empirical predictions that overlap to a large degree. Here, the focus is on their similarities, rather than on the differences (for a comparison among the three approaches, see Rigoli, 2019). To highlight the key logic shared by theories of referent-dependent evaluation, we will rely on a recent model proposed to integrate them (Rigoli, 2019; Rigoli et al., 2021; Rigoli & Martinelli, 2021; Woodford, 2012). The model proposes that the brain parcels out experience according to the context where this experience occurs (e.g., at school, at home, or in the workplace). When a new stimulus R (described by a real number reflecting an objective quantity such as a mark at school) is encountered in context *c* (e.g., at school), the proposal is that the brain estimates the stimulus’ subjective value V(R) as follows:

1 $$V(R)= logistic\ \left(\frac{R-{\mu}_c}{\sigma_c}\right)$$

Where *logistic* corresponds to a logistic function, implying that the equation also can be written as:

2 $$V(R)=\frac{1}{1-{e}^{-\frac{R-{\mu}_c}{\sigma_c}}}$$

**Fig. 1:**
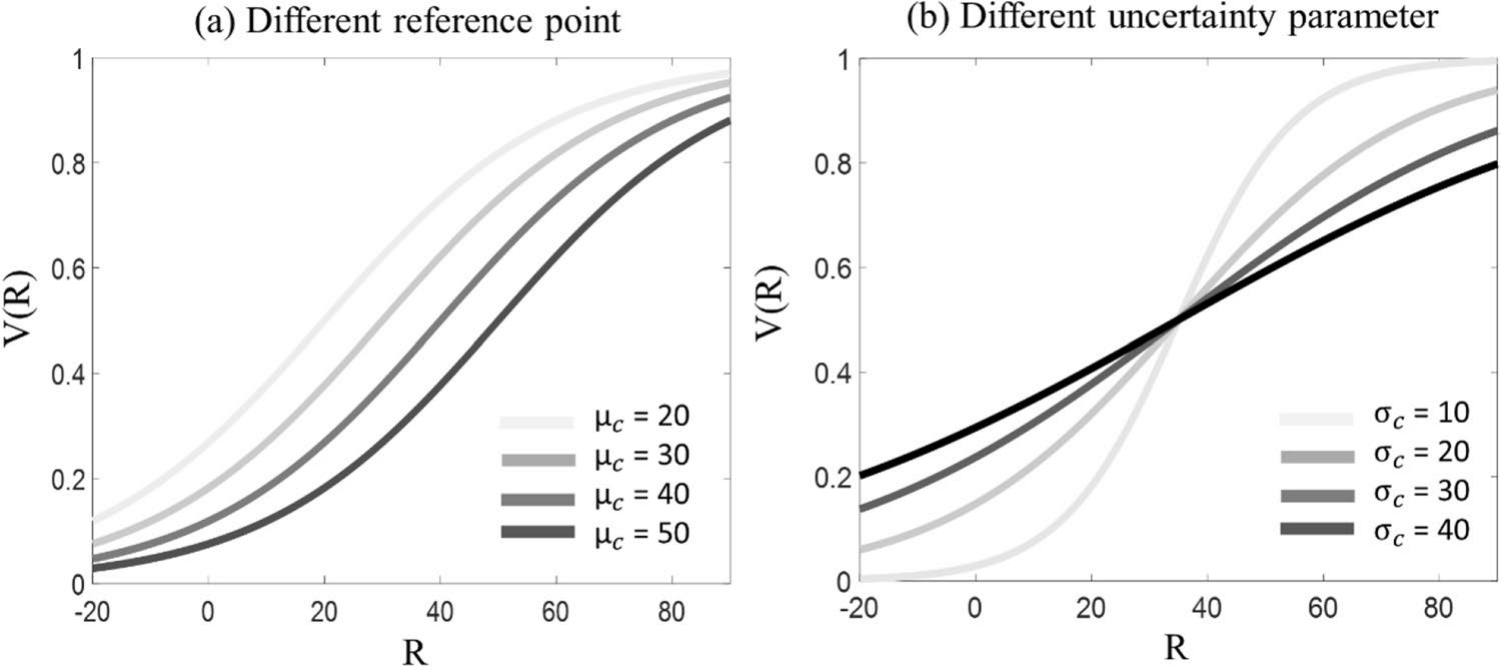
**Model parameters**

The parameter *μ*_*c*_ corresponds to a reference point to which the stimulus is compared (Figure 1A; note that each context has its own reference point parameter). Normally, this corresponds to the average of the contextual distribution (e.g., the distribution of stimuli encountered within the context) (Rigoli, 2019). The parameter *σ*_*c*_ captures the uncertainty about the context and usually corresponds to the standard deviation of the contextual distribution (Figure 1B; a more variable distribution is associated with higher uncertainty; note that each context has its own uncertainty parameter) (Rigoli, 2019). The uncertainty parameter *σ*_*c*_ can be interpreted as capturing the sensitivity to discrepancies between the stimulus R and the reference point *μ*_*c*_. In other words, it magnifies (when *σ*_*c*_ is small) or minimises (when *σ*_*c*_ is big) the difference between the stimulus R and the reference point *μ*_*c*_: when *σ*_*c*_ is small, the same difference between R and *μ*_*c*_ will be perceived as larger compared to when *σ*_*c*_ is big.

To summarise, contemporary theories of evaluation converge on the idea that the distribution of stimuli encountered in the past (the context) is critical during evaluation processes. These theories can be integrated within a model where two parameters reflect the reference point (usually the average of the contextual distribution) and the uncertainty (usually the standard deviation of the contextual distribution), respectively. Put it simply, the model proposes that subjective value corresponds to a z-score transformed by a logistic function (the latter implying that the result is bounded between zero and one). Below, we will explore how this model can be adopted to interpret addiction.

### Referent-dependent model of addiction

Let us attempt to adopt the framework introduced above to develop a referent-dependent model of addiction (RDMA). To illustrate the model, consider an example where, before addiction arises, a person associates a given context (e.g., home) with a distribution of stimuli (e.g., activities to do at home) whose average R is equal to 40 and standard deviation is equal to 20. The person will describe the home context adopting *μ*_*c*_ = 40 (capturing the average) and *σ*_*c*_ = 20 (capturing the standard deviation) as parameters. Consider two stimuli associated with the home context such as cleaning the house and having a family meal, linked with *R*_*CL*_ = 35 and *R*_*FM*_ = 45, respectively: according to equation 1, their subjective value will be V(*R*_*CL*_) = 0.44 and V(*R*_*FM*_) = 0.56, respectively. Imagine also that consumption of an addictive drug (e.g., heroin) is associated with this context. We propose to describe the experience of consuming an addictive drug as being associated with a stimulus R (in this example, *R*_*D*_ = 90, implying a subjective value of V(*R*_*D*_) = 0.92) which is much larger than any other stimulus associated with the context.

**Fig. 2:**
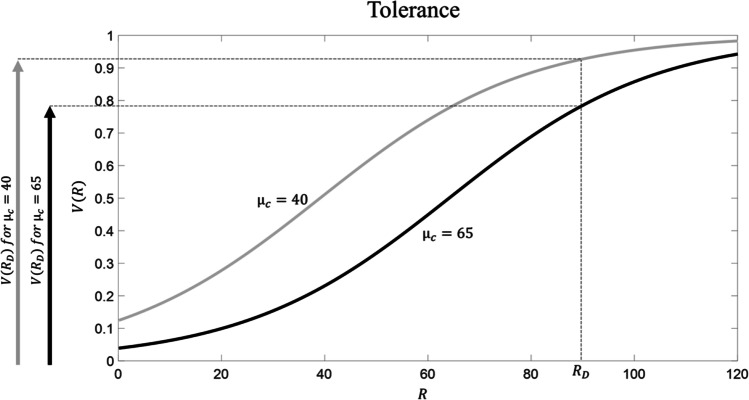
**Tolerance according to RDMA.** Before addiction arises, *μ*_*c*_ = 40 (in grey); addiction is associated with *μ*_*c*_ = 65 (in black). Drug consumption is associated with *R*_*D*_ = 90

**Fig. 3:**
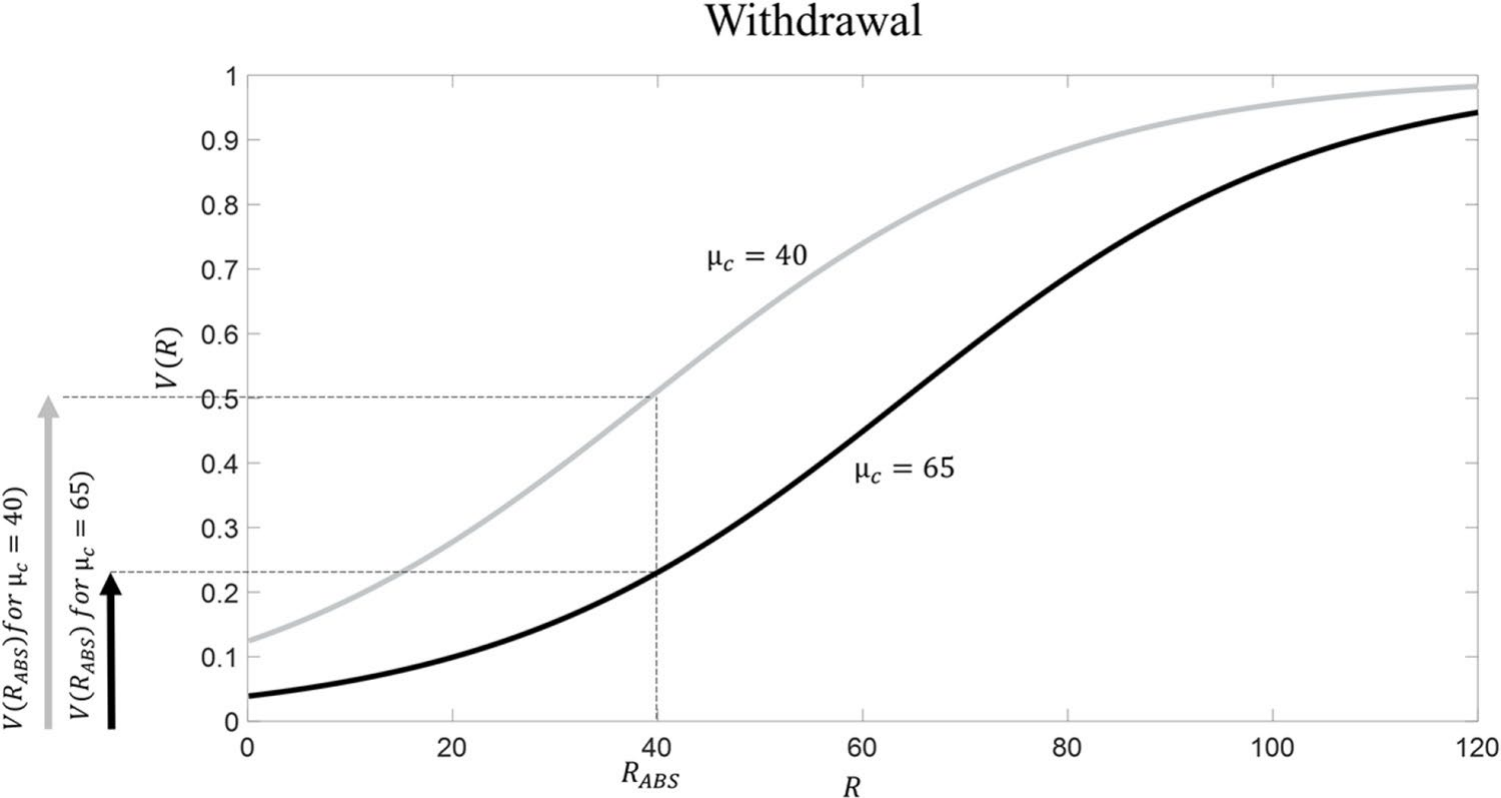
**Withdrawal according to RDMA.** Before addiction arises, *μ*_*c*_ = 40 (in grey); addiction is associated with *μ*_*c*_ = 65 (in black). Abstinence is associated with *R*_*ABS*_ = 40

**Fig. 4:**
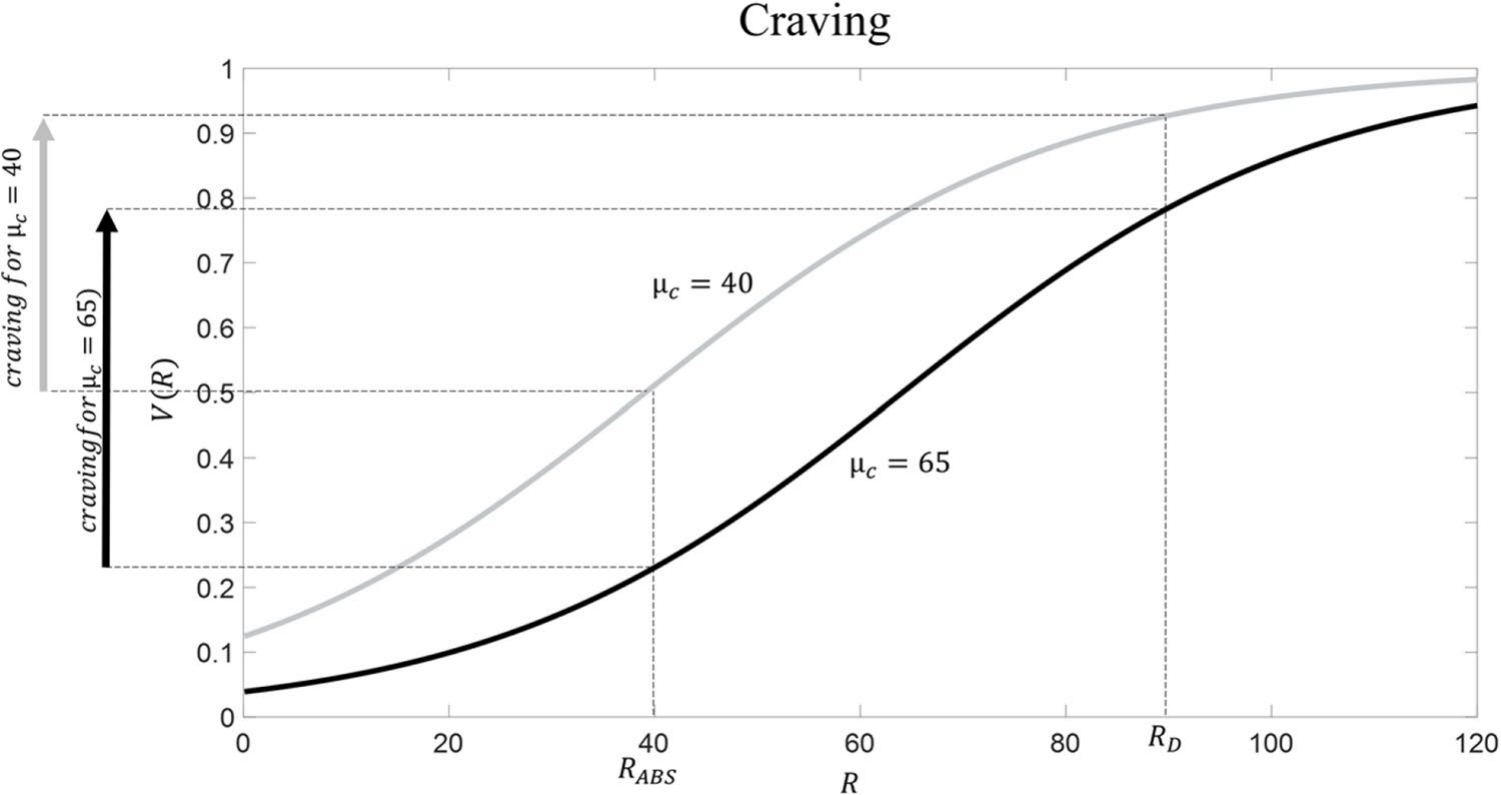
**Craving according to RDMA.** Before addiction arises, *μ*_*c*_ = 40 (in grey); addiction is associated with *μ*_*c*_ = 65 (in black). Drug consumption is associated with *R*_*D*_ = 90 and abstinence is associated with *R*_*ABS*_ = 40

What happens when the drug is consumed repeatedly over time? Because drug consumption is associated with very large R (being *R*_*D*_ = 90), repeated drug consumption increases the average R experienced in the context. According to the RDMA, this will boost the value of the reference point parameter *μ*_*C*_, because, as explained above, this parameter reflects the contextual average. For example, let us imagine that repeated drug consumption eventually leads to *μ*_*c*_ = 65. The question of which processes are responsible for an increased reference point is not the focus of the paper (it requires to examine how parameters are shaped by experience – see Discussion); it suffices to say that, according to the RDMA, an enhanced reference point *μ*_*C*_ is the result of repeated drug consumption. This idea has obvious analogies with set point theories of addiction (Ahmed & Koob, 1998; Keramati et al., 2017; Koob & Le Moal, 2001); below, we will examine similarities and differences between the RDMA and these theories. What are the consequences of having a reference point equal to *μ*_*c*_ = 65? We highlight five key consequences:

1. Tolerance (Figure 2): the subjective value of drug consumption is now much lower than before (V(*R*_*D*_) = 0.92 and V(*R*_*D*_) = 0.78 with *μ*_*c*_ = 40 and *μ*_*c*_ = 65, respectively). This implies that a larger dose of the drug (i.e., associated with R = 115) is necessary for eliciting the same subjective value. This aspect captures the phenomenon of drug tolerance, namely the fact that, when addiction emerges, the same initial drug dose is insufficient to produce the same effect as at the beginning (Turton & Lingford-Hughes, 2016).
2. Withdrawal (Figure 3). Let us assume that the condition of abstinence from the drug is, both before addiction and when addiction emerges, associated with *R*_*ABS*_ = 40; this number is proposed because, before addiction develops (i.e., when *μ*_*c*_ = 40), it is associated with a neutral state (occurring when V(R) = 0.5). The subjective value associated with abstinence corresponds to V(*R*_*ABS*_) = 0.5 and V(*R*_*ABS*_) = 0.22 with *μ*_*c*_ = 40 and *μ*_*c*_ = 65, respectively. In other words, at the beginning the subjective value of drug abstinence was neutral, but after repeated drug consumption it has now become bad (in general, a stimulus R can be considered as being bad when V(R) < 0.5). This aspect captures the phenomenon of withdrawal, namely the fact that, when addiction emerges, drug abstinence elicits a very unpleasant psychophysiological state (West & Gossop, 1994).
3. Increased craving (Figure 4). The notion of craving describes the desire for the drug (or the level of “wanting”; Robinson & Berridge, 1993), and thus it is a key determinant of the motivation for drug consumption (Skinner & Aubin, 2010; Tiffany & Wray, 2012). We propose a definition of *drug craving* as being equal to the difference in subjective value between drug consumption and abstinence from the drug:

3 $$drug\ craving=V\left({R}_D\right)-V\left({R}_{ABS}\right)$$

Our definition implies that the level of craving experienced in a certain context (e.g., at home) does not depend on the drug’s subjective value alone, but on comparing the drug’s subjective value against abstinence. This is inspired by theoretical models that interpret the motivation for performing an action (e.g., drug consumption) as dependent on the difference in subjective value between performing versus not performing the action (in our case, the difference between taking the drug and not taking the drug) (Dayan, 2012; Seligman, 1974). While, according to equation 3, craving is equal to 0.42 when *μ*_*c*_ = 40 (i.e., before addiction is established)), it reaches a level of 0.56 when *μ*_*c*_ = 65 (when addiction has developed). The reason is that, compared with abstinence, the subjective value of drug consumption is much higher during addiction. This is consistent with the observation that drug craving is boosted when addiction is established (Skinner and Aubin, 2010; Tiffany & Wray, 2012).

**Fig. 5:**
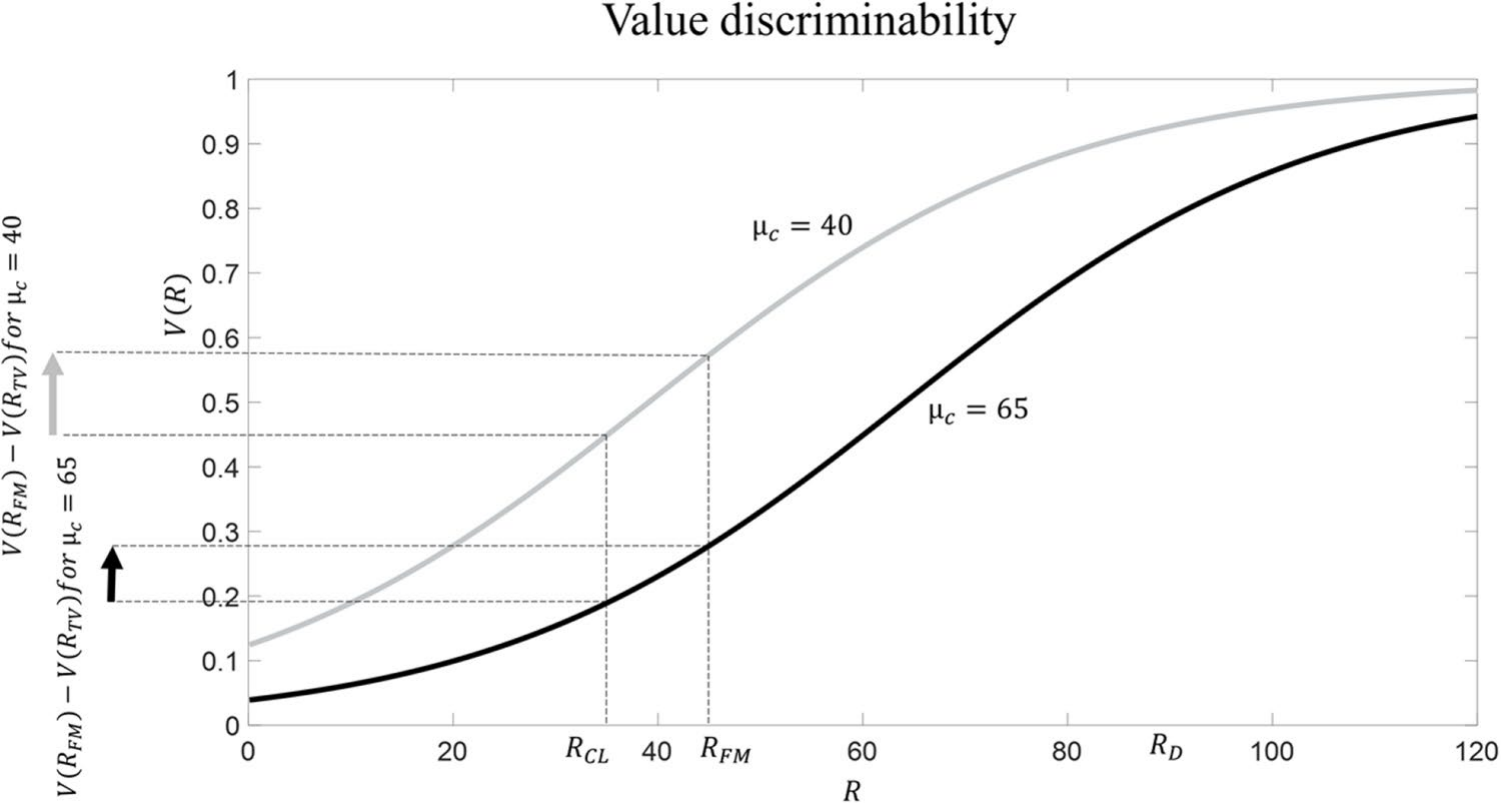
**Diminished value discriminability according to RDMA.** Before addiction arises, *μ*_*c*_ = 40 (in grey); addiction is associated with *μ*_*c*_ = 65 (in black). Cleaning the house is associated with *R*_*CL*_ = 35 and having a family meal is associated with *R*_*FM*_ = 45

4) Negative mood. An exaggerated reference point parameter *μ*_*c*_ (in our example equal to 65) implies that, compared with the initial reference point (in our example equal to 40), a much larger number of stimuli will be evaluated as being bad (in general, a stimulus R can be considered as being bad when V(R) < 0.5). In our example, while with *μ*_*c*_ = 40 cleaning the house (associated with *R*_*CL*_ = 35) was perceived as mildly bad but having a family meal (associated with *R*_*FM*_ = 45) was perceived as good, with *μ*_*c*_ = 65 now both stimuli are appraised as quite bad. This fits with studies showing that addicted patients often report a failure to enjoy normal everyday experiences and feel persistent negative mood (Destoop et al., 2019).
5) Diminished stimulus discriminability (Figure 5). The ability to discriminate between ecological stimuli has now diminished. Consider the difference in subjective value between cleaning the house and having a family meal: while this was equal to 0.12 when *μ*_*c*_ = 40, it has now diminished to 0.09 with *μ*_*c*_ = 65. This implies that, when *μ*_*c*_ = 65, one will be less careful when having to choose between cleaning the house and having a family meal. This aspect is compatible with the observation that addiction is often characterised by poor decision-making even when decisions do not involve any drug (Clark & Robbins, 2002; Koffarnus & Kaplan, 2018; Verdejo-Garcia et al., 2018).

Overall, the five points just described mimic fundamental aspects of addiction. Thus, the RDMA proposes that addiction can be explained by an excessive reference point *μ*_*c*_, which is produced by repeated experience with the drug.

Once the RDMA has been explored with respect to how addiction develops, it is critical to examine the model with respect to the question of how addiction can be treated. A large body of evidence indicates that detoxification (often achieved after a progressive diminution of the drug dose) followed by prolonged abstinence is beneficial for treating addiction (Diaper et al., 2014). According to the RDMA, what is the consequence of prolonged drug abstinence? The RDMA predicts that abstaining from the drug will be initially experienced as highly distressing, in line with the empirical observation of withdrawal symptoms (West & Gossop, 1994). However, if abstinence continues, the RDMA also implies that the reference point will progressively decrease, thus diminishing the severity of withdrawal symptoms. With sufficient time, the prediction is that the reference point will go back to normal (in our example corresponding to *μ*_*c*_ = 40), leading to great improvements in symptoms. This fits with evidence showing that prolonged drug abstinence appears to offer benefits to patients (Diaper et al., 2014).

However, evidence also demonstrates that, in many cases, drug abstinence is far from being resolutive (O'Brien, 2003; O’Brien et al., 1992). An issue often reported by patients is that, after months or even years of abstinence, they are likely to experience a sudden drug craving when reexposed to drug-related cues (Brownell et al., 1986; Marlatt, 1996). Evidence suggests that this delayed craving is a major factor for relapse, not rarely leading to a new cycle of prolonged drug use (O’Brien et al., 1992; Sinha, 2011). How can the RDMA explain craving occurring after a prolonged period of abstinence? The answer is that, while the RDMA assumes that one single context is activated each time, latent representations of multiple contexts are postulated to coexist in the brain. This idea has critical repercussions when explaining how addiction arises and how it can be treated. Regarding how addiction arises, this implies that addiction might not concern all available contexts, but only those where drug consumption repeatedly occurs (thus boosting the reference point *μ*_*c*_ of those contexts). Some life contexts (e.g., when being at the church) might remain relatively unaffected by addiction; in other words, they might remain characterised by a rather normal reference point *μ*_*c*_, implicating that less or even no symptoms will arise in those contexts. This aspect fits with empirical evidence indicating that addiction’s symptoms, such as craving and withdrawal are more likely to arise in certain contexts rather than others (Siegel, 2005). Regarding how addiction can be treated, the implication is that for a full recovery, all contexts where the reference point *μ*_*c*_ has been altered by drug use need to experience prolonged abstinence; this is to allow the reference point *μ*_*c*_ of all contexts to go back to normal. If an altered context is never engaged during prolonged abstinence, its reference point *μ*_*c*_ will remain excessive, and symptoms will arise when the context is finally activated (even if this occurs after a long period without consuming the drug). This can explain why, despite years of abstinence, delayed craving often arises after exposure to drug-related cues, that is, to cues activating an altered context that has remained unscathed by abstinence (Brownell et al., 1986; Marlatt, 1996).

In summary, this section introduces the RDMA as an attempt to explore the implications of referent-dependent evaluation to explain addiction. The model interprets addiction as a disorder characterised by an excessive reference point that arises because of repeated drug consumption. Five key elements of addiction ensue from this notion, including tolerance, withdrawal, increased craving, negative mood, and diminished stimulus discriminability. Moreover, the RDMA implies that addiction is context-specific, an aspect with important implications for explaining how addiction emerges and how it can be treated. The next section compares the picture offered by the RDMA with classical theories of addiction and assesses whether any new insight can be highlighted.

### Comparison with classical theories of addiction

The number of theories of addiction is enormous, and it is impossible to examine each one here. Thus, we will restrict the focus only on two broad families of theories, which are among the most influential in the literature. These include set point models (Ahmed & Koob, 1998; Keramati et al., 2017; Koob & Le Moal, 2001) and associative learning models (Di Chiara, 1999; Redish et al., 2007; Siegel, 2005). Both families will be discussed with regard to the RDMA in what follows.

Although they present important differences, all set point models are grounded on the notion that addiction arises from alterations of a set point representation in the brain (Ahmed & Koob, 1998; Keramati et al., 2017; Koob & Le Moal, 2001). The idea is that consumption of or abstinence from a drug produces effects that depend on a comparison with a set point. Before addiction develops, the set point is supposed to be at a low level, implying that a small drug dose is sufficient to produce euphoria, whereas abstinence from the drug is expected to have no effect. Repeated drug consumption is proposed to increase the set point. Tolerance to the drug is interpreted as being a consequence of such enhanced set point, implying that a small drug dose is now insufficient to produce the same euphoric effects and that a larger dose is required for these effects to emerge. Withdrawal also is produced, because when the set point is excessively high, abstinence from the drug is experienced as a very negative state. The similarity between set points models and the RDMA is remarkable, so much so that it seems reasonable to classify the RDMA as a version of set point models. Like the latter, the RDMA proposes that consumption of and abstinence from the drug produce effects that depend on a comparison with a form of set point, in this case corresponding to the referent point parameter *μ*_*c*_. On this basis, the explanation of tolerance and withdrawal proposed by the RDMA is essentially equivalent to the one ensuing from set point models.

However, we note that the RDMA appears to extend classical set point models in at least three aspects. First, as examined earlier, the RDMA implies that craving is stronger during addiction compared with before addiction. This is because the value function proposed by the RDMA is nonlinear (specifically, it is a logistic function), implying that the difference in subjective value between two stimuli will change depending on the reference point parameter *μ*_*c*_. Because the latter parameter increases when addiction develops, the difference in subjective value between drug consumption and abstinence from the drug, which is how we have defined craving, becomes larger during addiction compared with before addiction. On the contrary, standard set point models commonly assume a linear value function, implying that the difference in subjective value between two stimuli remains constant even when the set point changes (Ahmed & Koob, 1998; Keramati et al., 2017; Koob & Le Moal, 2001). It follows that, according to set point models, the difference in subjective value between drug consumption and abstinence, which is how we have defined craving, remains unaltered during addiction.

The second aspect distinguishing the RDMA from standard set point models also depends on the idea that, in the former, craving increases during addiction but now combined with the idea that addiction is context-specific. In other words, the RDMA postulates that the brain represents different contexts and describes each with a specific reference point parameter *μ*_*c*_. This entails that addiction development is context specific, namely that drug abuse can increase the reference point *μ*_*c*_ in some contexts but not in others, implying that symptoms will be manifested in some contexts but not in others (Siegel, 2005). Moreover, this can explain why craving can suddenly arise after prolonged abstinence (Brownell et al., 1986; Marlatt, 1996); this is interpreted as the consequence of being exposed to a context associated with an altered reference point parameter *μ*_*c*_. Contrary to the RDMA, classical set point models do not propose that addiction is context specific; instead, they presuppose that the same set point applies everywhere. Thus, they struggle to explain why addiction symptoms are more likely to occur in certain contexts. Moreover, they fail to explain why craving can emerge after prolonged periods of abstinence; in their view, prolonged abstinence should reestablish the original set point (which is the same for all contexts), thus preventing craving to occur.

A third implication of the RDMA neglected by classical set point models concerns the value attributed to stimuli unrelated with the drug. The RDMA predicts that addiction will be associated with diminished stimulus discriminability. In other words, it predicts that, when addiction develops, ecological stimuli will be perceived as more similar to one another in terms of subjective value. In our example above, addiction impairs the ability to discriminate between having a family meal (a better stimulus) and cleaning the house. Implications of this aspect encompass impairments during decisions where the drug is not at stake (Clark & Robbins, 2002; Koffarnus & Kaplan, 2018; Verdejo-Garcia et al., 2018). Again, this occurs because the RDMA proposes a nonlinear value function prescribing that the difference in subjective value between two stimuli will change depending on the reference point parameter *μ*_*c*_. Assuming a linear value-function, standard set point models do not predict any diminished value discriminability impairment during addiction; the difference in subjective value between two stimuli remains unaltered when the set point changes.

Let us examine the link between the RDMA and associative learning models. The latter propose that addiction develops because repeated drug consumption establishes an association between certain contexts and the drug (Di Chiara, 1999; Redish et al., 2007; Siegel, 2005). According to this view, the consequence of this association is that every time one is exposed to drug-related contexts, craving arises. Associative learning models nicely explain why drug craving is more likely to arise in drug-related contexts and when addiction is well established (Siegel, 2005). Moreover, these models provide an explanation of why craving can appear even after prolonged abstinence (Brownell et al., 1986; Marlatt, 1996). Abstinence might leave the association between a certain context and the drug intact, implying that experiencing that context will elicit craving even after prolonged abstinence. Although associative learning theories provide a compelling interpretation of the role of context, they struggle to account for other key aspects of addiction. First, tolerance and withdrawal are hard to reconcile with these theories. Second, it is unclear how these theories can explain why addiction is linked with diminished ability to discriminate among stimuli unrelated with the drug (in our example above, cleaning the house and having a family meal), because, according to associative learning theories, the value attributed to these stimuli does not change during addiction. Thus, the RDMA integrates associative learning models by offering a framework that can explain not only the role of context but also other phenomena, such as tolerance, withdrawal, and decreased stimulus discriminability.

Overall, our examination suggests a remarkable overlap between classical theories of addiction and the RDMA. This is true especially for set-point theories, as suggested by the analogy between the idea of set point and reference point. However, the RDMA resembles also associative learning models in the way it treats the role of context in addiction. Interestingly, the RDMA might offer a framework to reconcile set point models, well suited to explain phenomena such as tolerance and withdrawal, with associative learning models, particularly insightful to account for the role of context. While here we have focused on theoretical considerations, the next section examines the RDMA in the context of the neurobiology of addiction.

### Implications for the neurobiology of addiction

It is important to assess whether the picture offered by the RDMA is compatible with the neurobiology of addiction. To examine this, we will focus on the dopaminergic activity in the striatum of the basal ganglia, because this signal is known to be at the core of evaluation processes in the brain (Bartra et al., 2013; Schultz et al., 2015; Wise, 2004) and because alterations of this signal appear also to be critical in addiction (Willuhn et al., 2010; Wise & Robble, 2020).

It is well documented that, when a reward is experienced, striatal dopaminergic response reflects the reward’s subjective value (Bartra et al., 2013; Schultz et al., 2015; Wise, 2004). This also has been observed for addictive drugs (Willuhn et al., 2010; Wise & Robble, 2020). For example, data show that the striatal dopaminergic response to administration of an addictive drug correlates with the dose of the drug (this has been observed for a variety of substances; Willuhn et al., 2010; Wise & Robble, 2020). Moreover, at least for some substances (Nutt et al., 2015), neuroimaging studies in humans have found that the striatal dopaminergic response to an addictive drug correlates with subjective ratings about the associated level of euphoria (Laruelle et al., 1995; Volkow et al., 1999). As examined earlier, the RDMA implies that, keeping the dose constant, the subjective value of a substance will diminish as addiction develops. Applying this logic to the striatum, the prediction is that the striatal dopaminergic response to drug administration will decrease as addiction develops. This fits with several lines of evidence. Allowing rats to self-administer cocaine daily for 3 weeks, one study observed that these animals progressively increased drug consumption over time (a processes interpreted as mimicking addiction in humans) (Willuhn et al., 2014). As time elapsed, the phasic dopaminergic response to cocaine diminished in the striatum, an effect that was particularly strong in those animals showing higher day-by-day increase in drug consumption. Moreover, administration of the dopamine precursor L-DOPA counteracted the dopaminergic depletion and the increased consumption, in line with the possibility of a causal relation between dopaminergic response and consumption. Consistent findings have emerged from neuroimaging studies in humans. The first of these studies found that, compared with healthy controls, cocaine-dependent participants manifested diminished dopamine release in the striatum when administered with cocaine (Volkow et al., 1997). Another study showed that, compared with healthy controls, cocaine-dependent participants manifested diminished dopamine release in the striatum also when amphetamine was administered (Martinez et al., 2007). This was integrated by data indicating that this effect was weakened in cocaine-dependent patients who responded to treatment, but not in patients who did not respond (Martinez et al., 2011). A consistent picture emerges when considering what happens during withdrawal from the drug. As examined, during withdrawal the RDMA implies that a very adverse subjective value is experienced. The ensuing prediction is that during withdrawal the striatal release of dopamine will be inhibited. Empirical data broadly support this, both when considering tonic and phasic dopamine release, although mixed findings have emerged for psychostimulants (Stuber et al., 2005; Willuhn et al., 2010).

Evidence indicates that striatal dopamine response is not only elicited by drug consumption but also by exposure to drug-related contexts (Leyton & Vezina, 2013; Samaha et al., 2021; Willuhn et al., 2010). Specifically, the data reveal a dopaminergic increase when drug-related cues are experienced. The RDMA can explain this by interpreting the dopaminergic response to drug-related contexts as signalling craving. According to the RDMA, when addiction is established, drug-related cues activate a context representation where, because of an exaggerated reference point parameter *μ*_*c*_, craving is enhanced. At the neural level, this enhanced craving might be reflected in the striatal dopaminergic response. This possibility is supported by substantial evidence indicating that presentation of drug-related cues elicits craving and drug-seeking behaviour (Stuber et al., 2005;Volkow et al., 2006 ; Weiss et al., 2000). Even more compellingly, evidence demonstrates that the very same cues can trigger both dopamine release and craving/drug seeking (Volkow et al., 2006; Weiss et al., 2000). For example, a human neuroimaging study on cocaine-dependent participants examined striatal dopaminergic activity comparing a group exposed to a cocaine-related video against a group presented with a neutral video (Volkow et al., 2006). Not only higher striatal response was found for the cocaine-related video group, but this response also correlated with the level of craving reported by participants during the video.

Altogether, empirical evidence about the role of the striatal dopaminergic signal in addiction, which is a crucial element of the disorder’s neurobiology, appears to be broadly consistent with the RDMA. Specifically, the RDMA fits with the observations that (i) addiction reduces the dopaminergic response in the striatum to the substance, (ii) withdrawal is associated with dopaminergic inhibition, (iii) and, when addiction is established, exposure to drug-related contexts triggers dopamine release, which underlies craving. According to the RDMA, all of these phenomena are ultimately the expression of a single factor, that is, of an exaggerated reference point parameter *μ*_*c*_. An intriguing possibility is that, at the neural level, this factor corresponds to the number of D2 receptors available in the dopaminergic circuit (with higher number of receptors reflecting lower reference point parameter *μ*_*c*_). This possibility is supported by evidence showing that, for a variety of substances investigated, a decreased number of these receptors is observed in addicted patients (Volkow et al., 2009).

## Discussion

Because impaired evaluation processes are central to addiction and because reference dependency is at the core of evaluation, this paper asks whether any insight can be afforded by interpreting addiction as a form of maladaptive referent-dependent evaluation—a question we address by introducing the RDMA. Our analysis suggests that the RDMA has some plausibility, given that several key aspects of addiction can be derived, so to speak, automatically by adopting this outlook. In other words, withdrawal, tolerance, enhanced craving, negative mood, and diminished stimulus discriminability all ensue by postulating a referent-dependent evaluation framework without adding any further assumption. Moreover, the pattern of striatal dopamine activity observed in addiction appears to be broadly consistent with predictions ensuing from the RDMA (Willuhn et al., 2010). Of note is the consideration that classical accounts of addiction, such as set-point theories (Ahmed & Koob, 1998; Keramati et al., 2017; Koob & Le Moal, 2001) and associative learning theories (Di Chiara, 1999; Redish et al., 2007; Siegel, 2005) share a great deal with the RDMA. Overall, this encourages research to consider the referent-dependent evaluation framework as a useful add-on to the theoretical toolkit adopted to interpret addiction and to inform future empirical work.

The RDMA makes novel predictions that can inspire empirical research. Some of these arise from the idea that addiction is accompanied by diminished stimulus discriminability. Broadly speaking, this implies impaired decision-making even when the drug is not at stake, which is in line with empirical evidence (Clark & Robbins, 2002; Koffarnus & Kaplan, 2018; Verdejo-Garcia et al., 2018). However, diminished stimulus discriminability is a more specific concept: it predicts that addicted patients will exhibit lowered value sensitivity during choice, namely that their choices will depend to a lesser degree on the values at stake. To understand what this means, take the choice between cleaning the house (linked with R = 35) and having a family meal (linked with R = 45) in our example above. Assuming (as many decision models do; Luce, 1959) that the choice probability for each option depends on the difference in subjective value between options, imagine that, before addiction develops, the family meal is chosen 80% of the times. The RDMA implies that, when addiction is established, the difference in subjective value between the two options will diminish, implying that now the family meal will be chosen, say, only 60% of the times. Moreover, as Figure 5 illustrates, the RDMA predicts that, in addiction, diminished stimulus discriminability is not constant, but it is more pronounced in the domain of punishment (in this example, for stimuli associated with R < 40, being the initial reference point equal to 40) compared to the domain of reward (in this example, for stimuli associated with R > 40). This predicts that addiction will produce lowered value sensitivity when choosing among punishments compared to when choosing among rewards. Testing RDMA’s predictions concerning stimulus discriminability appears as a promising research endeavour. To this aim, research can adopt established paradigms in the literature that have been developed specifically to assess value sensitivity (Martinelli et al., 2018).

Above, we have briefly overviewed contemporary theories of referent-dependent evaluation, including decision-by-sampling (Stewart et al., 2006), divisive normalization theory (Louie et al., 2015), and expectation-as-reference models (Kőszegi & Rabin, 2006; Rigoli et al., 2016). To the list, it is worth adding range-frequency theory, a general account of context effects at play in various domains beyond evaluation (Parducci, 1965). It is not the purpose of the paper to compare the different approaches but to stress their similarities. Yet, although a systematic comparison among the different approaches requires further investigation, a recent paper (Rigoli, 2019) suggests that when evidence about context effects elicited by previous rewards (which is the scenario relevant in the domain of addiction) is assessed, the model employed by the RDMA outperforms other accounts.

The RDMA may be relevant when considering the issue of treatment. Broadly speaking, the theory implies that abstinence during exposure to drug-related contexts should benefit patients. Shared also by classical conditioning models, this idea has inspired a number of clinical protocols collectively known as Cue Exposure Therapy (Drummond et al., 1995). Empirical evidence suggests that this is to some extent effective but also that it does not outperform alternative approaches, therefore remaining nonstandard in clinical practice (Kiyak et al., 2022; Martin et al., 2010; Mellentin et al., 2017). Yet, there is scope for improvement (Conklin & Tiffany, 2002), and the RDMA may provide some clues. Although this is not the place for a detailed discussion, the RDMA encourages clinicians to consider the following possibilities: (i) covering a large number of contexts, (ii) ensuring prolonged exposure to each, (iii) targeting not only single cues (e.g., images of a syringe), but also broader environments (e.g., when joining a party or when experiencing a certain mood), (iv) tailoring the intervention to the specific contexts experienced by each patient.

Although, in its current form, the RDMA is able to accommodate several key aspects of addiction, understanding other facets of the disorder requires to extend the theory further. First, we have not examined the question of how precisely repeated drug consumption produces an exaggerated reference point. To address this, it is necessary to examine how, within the RDMA framework, parameters are shaped by experience. How this occurs is the focus of various reinforcement learning models; integrating these with the RDMA appears as a promising research avenue (Palminteri et al., 2015; Rigoli et al., 2018).

A related question is what happens when an addicted patient encounters a new context. In the language of the RDMA, the question is which parameters are attributed to a new context. A possibility is that the parameters of a new context are not abnormal, thus producing no symptoms. Alternatively, they may correspond to an average across previous contexts (or across contexts similar to the new one), thus producing symptoms. This aspect remains to be examined empirically. Moreover, the concept of context employed by the theory requires further elaboration. While typically contexts refer to external cues or locations, these can also encompass internal cues, such as proprioceptive and emotional states associated with drug administration (Siegel, 2005). An interesting avenue is to employ the RDMA to study the role of such internal contexts.

Another question that remains unanswered by the RDMA is what happens when addiction becomes more and more established. Simply stated, at the moment the RDMA does not distinguish between addiction lasting for 1 year versus addiction lasting for 20 years. With this regard, there is evidence suggesting that, while goal-directed processes are prominent during the early phase of the disorder, habitual mechanisms prevail when addiction becomes deeply entrenched (Everitt & Robbins, 2005, 2016). Future work adopting the RDMA should ask how to consider the different role of goal-directed and habitual processes in addiction.

A limitation of the RDMA concerns the phenomenon of incubation of craving, that is, the finding that craving grows as abstinence unfolds up to a point when it progressively decreases (Grimm et al., 2001). In its current version, the RDMA implies that craving should progressively decrease with abstinence, because the reference point progressively decreases. However, craving incubation might be reconciled within the RDMA if one assumes that, as it unfolds, abstinence is not associated with the same stimulus. For example, in the scenario above abstinence is constantly associated with R = 40; a possibility is that, instead, the R of abstinence changes over time in such a way that it decreases as abstinence progresses, up to a point (e.g., R = 30) where it starts to grow and reaches R = 40 again. In any case, it is important to examine whether and how craving incubation can be reconciled with the RDMA.

As another limitation, the current version of the RDMA ignores individual differences (George & Koob, 2022). The more obvious example is that it ignores the fact that repeated drug consumption leads to addiction for some people but not for others. Considering individual differences is an important further step in the development of the RDMA.

It is important to analyse the RDMA not only vis-à-vis set point and classical conditioning models but also considering other influential perspectives. Among these, incentive sensitization theory is a prominent one (Robinson & Berridge, 1993). According to it, an exaggerated desire (wanting) of a substance, caused by dopaminergic alterations, is at the core of addiction. Both drug consumption and exposure to drug-related cues are proposed to elicit such uncontrollable desire, an effect which, according to the theory, typically persists even after prolonged abstinence. More than classical conditioning and set point accounts, incentive sensitization theory stresses the role of incentive value, a concept central also to the RDMA. Another aspect in common with the RDMA concerns the role attributed to cues as factors eliciting craving. Thus, overall, the two theories overlap to a substantial degree. The RDMA integrates incentive sensitization theory by examining the implications of addiction for stimuli unrelated with the drug, stressing the role of impaired discriminability.

When we overviewed evidence about dopaminergic response in the striatum, a broad consistency with the RDMA emerged. Nonetheless, it is important to highlight that the available neuroscientific data extend beyond the aspects presented above. To begin with, although the striatum and dopamine are important, they are only one component of the brain circuit involved in addiction. Other critical regions appear to be the orbitofrontal cortex (Schoenbaum & Shaham, 2008), the insula (Droutman et al., 2015), and the amygdala (See et al., 2003). A more complex picture emerges even when looking at the precise role of the striatum and dopamine. First, research has highlight different roles for the dorsal and ventral parts of the striatum, and, within the latter region, for the core and the shell of the nucleus accumbens (a subregion of the striatum) (Di Chiara, 2002). Second, evidence reveals a distinct role played by D1 and D2 dopaminergic receptors, both widely available in the striatum (Ashok et al., 2017; Kai et al., 2015). Third, in animals the direction of the dopaminergic response to a substance appears to vary based on the specific administration pattern employed in the experiment (Leyton & Vezina, 2013; Samaha et al., 2021). A full assessment of the consistency between the RDMA and the neuroscience of addiction requires a careful consideration of this complex picture.

One last limitation of the RDMA is that, in its current version, it does not distinguish between different addictive substances. Although the theory aims at offering a template for interpreting virtually all types of substance dependence, yet the physiological and psychological effects of different drugs vary greatly (Wise & Robble, 2020). For example, evidence shows that withdrawal symptoms are more severe in the case of opioids and alcohol compared to cocaine and nicotine. Future research employing the RDMA should consider such forms of variability across substances.

## Conclusions

In conclusion, this paper suggests that addiction can be fruitfully interpreted as a form of maladaptive referent-dependent evaluation. The contribution of this argument it twofold. First, it offers a new perspective to look at the disorder, potentially fostering further theoretical work and inspiring empirical investigations. Second, it adds to recent work supporting the idea that referent-dependent evaluation theory might offer a general framework to interpret forms of psychopathology where disrupted affective and motivational processes are critical (Rigoli et al., 2021; Rigoli & Martinelli, 2021).

## Data Availability

N/A
